# New Applications of HBOC-201: A 25-Year Review of the Literature

**DOI:** 10.3389/fmed.2021.794561

**Published:** 2021-12-08

**Authors:** Min Cao, Yong Zhao, Hongli He, Ruiming Yue, Lingai Pan, Huan Hu, Yingjie Ren, Qin Qin, Xueliang Yi, Tao Yin, Lina Ma, Dingding Zhang, Xiaobo Huang

**Affiliations:** ^1^Department of Critical Care Medicine, Sichuan Academy of Medical Sciences and Sichuan Provincial People's Hospital, University of Electronic Science and Technology of China, Chengdu, China; ^2^Anesthesiology, Southwest Medicine University, Luzhou, China; ^3^Surgical Department, Chengdu Second People's Hospital, Chengdu, China; ^4^Health Inspection and Quarantine, Chengdu Medical College, Chengdu, China; ^5^Sichuan Provincial Key Laboratory for Disease Gene Study, Sichuan Provincial People's Hospital, University of Electronic Science and Technology of China, Chengdu, China

**Keywords:** HBOC-201, red blood cell, oxygen-carrying capacities, oxygen bridge, clinical settings

## Abstract

If not cured promptly, tissue ischemia and hypoxia can cause serious consequences or even threaten the life of the patient. Hemoglobin-based oxygen carrier-201 (HBOC-201), bovine hemoglobin polymerized by glutaraldehyde and stored in a modified Ringer's lactic acid solution, has been investigated as a blood substitute for clinical use. HBOC-201 was approved in South Africa in 2001 to treat patients with low hemoglobin (Hb) levels when red blood cells (RBCs) are contraindicated, rejected, or unavailable. By promoting oxygen diffusion and convective oxygen delivery, HBOC-201 may act as a direct oxygen donor and increase oxygen transfer between RBCs and between RBCs and tissues. Therefore, HBOC-201 is gradually finding applications in treating various ischemic and hypoxic diseases including traumatic hemorrhagic shock, hemolysis, myocardial infarction, cardiopulmonary bypass, perioperative period, organ transplantation, etc. However, side effects such as vasoconstriction and elevated methemoglobin caused by HBOC-201 are major concerns in clinical applications because Hbs are not encapsulated by cell membranes. This study summarizes preclinical and clinical studies of HBOC-201 applied in various clinical scenarios, outlines the relevant mechanisms, highlights potential side effects and solutions, and discusses the application prospects. Randomized trials with large samples need to be further studied to better validate the efficacy, safety, and tolerability of HBOC-201 to the extent where patient-specific treatment strategies would be developed for various clinical scenarios to improve clinical outcomes.

## Introduction

Tissue ischemia and hypoxia cause increased anaerobic metabolism, ion imbalance, mitochondrial uncoupling, activation of endothelial cells and various cell death programs, and pro-inflammatory immune responses (1, 2). Timely resolution of ischemia and hypoxia in cells, tissues, and organs is beneficial in restoring the cellular demand for energy metabolism and oxygen. In clinical practices, the increase of red blood cells (RBCs) and hemoglobin (Hb) in the blood is promoted mainly by the use of erythropoiesis-stimulating agents (ESAs) (3), intravenous iron (4), vitamin B12 (5), and folic acid (6) to improve tissue oxygenation. In the case of life-threatening diseases requiring blood transfusion, allogeneic blood transfusion is the most common and closest physiological option to improve the blood volume, Hb, and hypoxia of the patient. However, blood has some potential disadvantages including immune phenomena, blood infection, cross-matching, and scarcity. Therefore, the search for ideal blood substitutes to provide oxygen to ischemic and hypoxic tissues and organs to maintain normal cellular energy metabolism is an ongoing quest.

Hemoglobin-based oxygen carrier-201 (HBOC-201) [hemoglobin glutamer-250 (bovine); Hemopure, HbO_2_ Therapeutics LLC, Souderton PA 18964, USA] is a solution of purified, glutaraldehyde-polymerized, stroma-free, bovine Hb (7). HBOC-201 has been on the market in South Africa since 2001 and is approved for surgical patients with acute anemia (8). However, in 2008, the meta-analysis of cell-free hemoglobin-based blood substitutes and risk of myocardial infarction (MI) and death by Natanson et al. and related editorials have caused widespread controversy over the safety of clinical patients using HBOC-21 (9–12). The meta-analysis included 13 randomized controlled trials and concluded that the use of HBOCs was associated with a significantly increased risk of death and MI based on analysis of available data (9). The Food and Drug Administration (FDA), hence, suspended all the HBOC trials in the United States. However, related editorials suggested that the conclusions of this meta-study based on mixed studies at different levels must be treated with caution (10–12). They argued that the imminent risk of death due to low Hb outweighs the risk of HBOCs, so indiscriminately requiring suspension of all the HBOC trials could be fatal to these patients. The appeal submitted by the company that produces HBOC-201 to the Drug Control Board was finally successful in 2010 and the company officially resumed the registration. HBOC-201 is an investigational product that is currently only available through Expanded Access (EA). The EA program has been conducted in the United States since 2014, primarily in patients who have refused blood transfusions due to their religious beliefs (13). It is also currently approved for veterinary use in the United States and the European Union (14). HBOC-201 serves as a temporary oxygen bridge to improve local and systemic oxygenation and as a substitute for effective resuscitation fluids and RBCs in emergencies (15, 16). HBOC-201 can be used in various ischemic and hypoxic clinical scenarios such as traumatic hemorrhagic shock, hemolysis, MI, cardiopulmonary bypass, perioperative period, and organ transplantation. Compared with various resuscitation fluids, HBOC-201 is fluid as a volume expander and retains the ability to bind and release oxygen. When RBCs are not available, HBOC-201 can also replace RBCs as an oxygen bridge to improve tissue oxygenation *in vivo* until enough RBCs are produced to meet the needs of life. The main adverse effects currently reported with HBOC-201 include vasoconstriction, MI, and increased methemoglobin, which can be managed with close clinical monitoring and appropriate therapeutic measures (17, 18).

The purpose of this study is to summarize the properties of HBOC-201, the application of HBOC-201-related preclinical and clinical studies in various clinical scenarios, adverse events (AEs) and corresponding solutions, and prospects for its application. In this study, all the HBOC-201-related preclinical studies and clinical studies searched in Pubmed were included. From the selected studies, we further extracted information on HBOC-201-related clinical trials (Table 1) and case reports (Table 2). By reviewing the relevant preclinical and clinical studies of HBOC-201, clinical decision-makers are familiar with the characteristics and indications of HBOC-201 that could benefit patients in emergencies.

**Table 1:** Summary of clinical trials of hemoglobin-based oxygen carrier-201 (HBOC-201).

**References**	**Blinding, study type**	**Clinical scenario**	**Dose of HBOC-201**	**Patients population**	**Outcome**	**Physiologic effects**	**Author conclusion**
Mackenzie et al. (13)	Unblinded, multicenter study	Life-threatening anemia	Loading dose (2–4 units); 1–2 units infusion every 19–20 h, or continuous infusion	HBOC-201 (*n* = 54)	Early use of HBOC-201 can improve the survival chance of patients with acute bleeding and hemolysis. HBOC-201 did not cause serious adverse events.	Hypertension (SBP = 160 mmHg), increased liver enzymes and methemoglobin	Early use of HBOC-201 can increase the survival chance of patients with acute bleeding and hemolysis. If the duration and degree of low hemoglobin before HBOC-201 treatment is minimized, the patient is more likely to survive.
Mackenzie et al. (15)	N	Acute anemia, untreatable anemia, when no blood is available, when blood cannot be given	N	HBOC-201 (*n* = 1,701)	When hemoglobin is <5 g/dL, early use of HBOC-201 can improve the patient's chance of survival. HBOC-201 transfusion avoidance of 96% for 24 h, 70% for 1 week. More non-serious events occurred in the HBOC-201 group. Age, history of heart disease, and Hb deficiency are predictors of cardiac ischemic events.	Increased blood pressure, oliguria, gastro-intestinal symptoms, yellow skin and scleral discoloration, decreased pulse oximetry measurements and transient increased in methemoglobin, hepatic and pancreatic enzymes.	HBOC-201 transfusion was well-tolerated. When blood is not available or cannot be provided, HBOC-201 should be considered, and can maintain O_2_ delivery. Infusion of HBOC-201 can save blood transfusion has been proven in Phase 3 clinical trials.
Serruys et al. (19)	Randomized 3-arm (1:1:1), double-blind, placebo-controlled, dose-finding pilot (phase II)	Unstable angina or NSTE ACS and had a severe stenosis in at least one coronary artery eligible for PCI	15 or 30 g	15 g HBOC-201 (*n* = 15); 30 g HBOC-201 (*n* = 15); non-oxygen carrier colloid (*n* = 15)	HBOC-201 produced an increase in SBP, pulmonary capillary wedge pressure and calculated SVR and a concomitant decrease in CO. The proportion of patients with adverse events did not differ significantly between the two groups.	Increased blood pressure, transient increased in liver transaminases and/or pancreatic enzymes, increased methaemoglobin andcell-free plasma hemoglobin	HBOC-201 had no effect on resting and hyperaemic coronary blood flow. No compromise in the coronary blood flow or LVSWI was observed despite HBOC-201's known vasoactive effects.
Meliga et al. (20)	single-center, phase II, placebo controlled, crossover, single-blind	coronary balloon occlusion	11–12 g/dl at 48 ml/min up to 3 min	N=5	EF, CO, and dP/dTMIN decreased significantly and the EDP and time constant of relaxation increased significantly during dry occlusions in the HBOC-201 group. HBOC-201 failed to alter conduit coronary artery tone	Increased blood pressure	Intracoronary infusion of oxygenated HBOC-201 is capable of preserving left ventricular function, likely through maintenance of myocardial oxygenation. HBOC-201 can effectively preserve myocardial mechanical and electrical properties in the face of total coronary occlusion.
Sprung et al. (21)	Randomized, single-blinded, multicenter study	surgery	0.6, 0.9, 1.2, 1.5, 2.0, or 2.5 g/kg of body weight	HBOC-201 (*n* = 42); Lactated Ringer's (*n* = 26)	HBOC-201 didn't reduce the use of blood during the entire hospitalization.	Serum transaminases, transient skin discoloration, increased methemoglobin	HBOC-201 was generally well-tolerated. A single dose of HBOC-201 did not reduce the intraoperative allogeneic blood requirements.
Levy et al. (22)	Randomized, double-blind efficacy trial	Cardiac surgery	Initial dose (60 g in 500 mL); two subsequent dose (30 g in 250 mL); extra demand replenishes RBC	HBOC-201 (*n* = 50); RBC (*n* = 48)	HBOC-201 eliminated the need for red blood cell transfusions in 34% of patients. Oxygen extraction was greater in the HBOC group. Hematocrit values were transiently lower in the HBOC group.	Hypertension	HBOC-201 may be an initial alternative to RBC transfusions for patients with moderate anemia after cardiac surgery.
Jahr et al. (23)	Randomized, single-blind, parallel-group multicenter study	Elective orthopedic surgery	Loading dose (65 g); up to an additional 260 g; extra demand replenishes PRBC	HBOC-201 (*n* = 35); PRBC (*n* = 33)	HBOC-201 eliminated the need for red blood cell transfusions in 59.4% of patients; Adverse events and serious adverse events have a higher incidence in HBOC-201.	Troponin, hypertension	Patients <80 years old with moderate clinical need may safely avoid transfusion when treated with up to 10 units of HBOC-201; Adverse events may be related to patient age, volume overload, undertreatment and was isolated to patients that could not be managed by HBOC-201 alone.
Van et al. (24)	Randomized, single-blind, multicenter study	Non-cardiac surgery	Initial dose [2 units (60 g Hb)];up to 7 units	HBOC-201 up to 7 units (*n* = 83); RBCs (*n* = 77)	HBOC-201 eliminated the need for red blood cell transfusions in 43% of patients. There were no significant differences in mortality and the incidence of serious adverse events.	Hypertension, fever, transient jaundice	The ability of HBOC-201 to restore total Hb was less than RBCs. The short-term effect of HBOC-201 transfusion on HCT was similar to that of plasma volume expanders, and on Hb it is similar to that of whole blood. In addition, the relatively short (19–24 h) half-life of HBOC-201 required constant re-dosing to maintain total Hb levels.
LaMuraglia et al. (25)	Randomized, single-blinded, multicenter study	Aortic surgery	Initial dose 60 g; three more doses (30 g each) within 96 h; extra demand replenishes RBC	HBOC-201 (*n* = 48); RBC (*n* = 24)	HBOC-201 eliminated the need for red blood cell transfusions in 27% of patients.	Hypertension, increased in serum urea nitrogen concentration	HBOC-201 transfusion was well-tolerated and did not influence morbidity or mortality rates. HBOC-201 may provide a temporary oxygen transport bridge until endogenous RBC can recover adequate oxygen-carrying capacity, especially when RBC are not immediately available but surgery is urgently required.
Kasper et al. (26)	Unblinded, randomized study	Elective abdominal aottic surgery	6.9 ml/kg or 9.2 mL/kg (HBOC-20113.4 +- 0.7 g/dL)	HBOC-201 (*n* = 12); 6% hydroxyethyl starch (HES) (*n* = 12)	HBOC-201 (0.9 and 1.2 g/kg hemoglobin) increased perioperative vascular resistance and reduce cardiac output.	Increased vascular resistance [systemic (SVRI) and pulmonary vascular resistance (PVRI)];decreased cardiac output	Bovine hemoglobin in a dose range of 55–97 g hemoglobin increases vascular resistance and reduces cardiac output in patients undergoing anesthesia. However, HBOC-201 has no obvious advantages in hemodynamics and oxygen transport compared with hydroxyethyl starch. It may be that the advantage of increased oxygen-carrying capacity is offset by increased vascular resistance and decreased cardiac output.
Dubé et al. (16)	Randomized, single blind, multi-center, phase III clinical trial	Elective orthopedic surgery	loading dose (2-unit);10 units received in total	HBOC-201 (*n* = 121); PRBCs (*n* = 115)	Hemoglobin deficit in patients treated with HBOC-201 was more common than in the PRBC control group and emerged as a predictor of SAEs in a logistics model. The subjects randomly assigned to HBOC-201 had more severe anemia, which may be the cause of more SAE in the HBOC group.	myocardial infarction; cardiotoxic	HBOC can serve as a bridge to meet the oxygen-carrying needs of patients with severe anemia until RBC is available or patients can establish a safe hematocrit. However, according to the principle of relative efficacy, this clinical trial had shown that blood or PRBCs are generally preferred treatment for severe anemia when available and acceptable to the patient.
Pearce et al. (27)	N	Surgery	N	HBOC-201 (*n* = 806); Control (*n* = 661)	The research supports that the application of HBOC-201 can be extended to the treatment and management of trauma and ischemia. Severely injured patients may urgently need early blood transfusion, especially when blood is scarce or unavailable. The oxygen delivery of HBOC201 can lifesaving in the pre-hospital environment.	anemia, tachycardia, abdominal pain, diarrhea, dysphagia, nausea, vomiting, pyrexia, jaundice, lipase increases, oliguria, and hypertension	HBOC-201 was well-tolerated in various doses and regimens, as well as in various clinical, particularly perioperative. As the dose is increased for a long time, it is proved that HBOC-201 may provide a temporary oxygen transport bridge until endogenous RBC mass can restore adequate oxygen-carrying capacity, especially when RBCs are not immediately available but surgery is urgently required.
van et al. (28)	Controlled trials	Liver transplantation	N	DHOPE-COR-NMP (*n* = 16);successfully transplanted (*n* = 11)	During NMP, all livers cleared lactic acid and produced sufficient bile volume. However, five livers was excluded from transplantation due to bile pH <7.45. 69% of discarded human livers meeting all criteria were successfully transplanted, with 100% patient and graft survival at 3 and 6 months.	N	In summary, this prospective clinical trial proved the safety and feasibility of combining sequential DHOPE-COR-NMP and HBOC-201 to transplant human high-risk liver transplantation. The pretransplant resuscitation and viability assessment of these discarded livers resulted in a 20% increase in the number of deceased donor liver transplants in author's center.
de Vries et al. (17)	N	Liver transplantation	N	DHOPE-COR-NMP (*n* = 7); successfully transplanted (*n* = 5)	DHOPE-COR-NMP using an HBOC-based perfusion fluid protected livers against ischemia-reperfusion injury and performed hepatobiliary viability assessment before transplantation. The graft survival rate at 3 months after transplantation was 100%.	Increased methemoglobin	The use of a novel sequential DHOPE-COR-NMP using an novel HBOC-201-based perfusate eliminated the need to change the perfusate at different temperature stages and offered a novel method of liver machine perfusion for combined resuscitation and viability testing of discarded livers before transplantation. HBOC-201 appeared to be a safe alternative for RBC as oxygen carrier in DHOPE-COR-NMP.

*N, not reported; SBP, systolic blood pressure; CO, cardiac output; LVSWI, left ventricular stroke work index; EF, ejection fraction; dP/dTMIN, minimal rate of LV pressure change; EDP, end-diastolic pressure; RBC, red blood cell; PRBC, packed red blood cell; Hb, hemoglobin; GI, gastrointestinal; SVR, systemic vascular resistance; PVR, pulmonary vascular resistance; FFP, fresh frozen plasma; HOPE, hypothermic oxygenated perfusion; COR, controlled oxygenated rewarming; NMP, normothermic machine perfusion; UW, University of Wisconsin*.

**Table 2:** Summary of cases report of HBOC-201.

**References**	**Study Type**	**Clinical scenario**	**Patients population**	**Dose**	**Outcome**	**Adverse event**	**Author conclusion**
Marinaro et al. (29)	Case report	Traumatic brain injury	Jehovah's Witness	Initial dose (4 units); 2 units for 2 days	Died	Hypertensive event (systolic to 280 mmHg)	HBOC-201 rapidly corrected cerebral venous, central venous oxygen saturation and profound anemia.
Mullon et al. (30)	Case report	Severe autoimmune hemolytic anemia	Acute hemolysis	The first unit 0.25 g per minute; Subsequent units 0.50 g per minute; a total of 11 units	Discharged	Average mean arterial pressure was 104.7 mm Hg, pulmonary arterial systolic and diastolic pressures increased slightly	HBOC-201 may support oxygen delivery in patients with severe autoimmune hemolytic anemia.
Gannon et al. (31)	Case report	Gastrointestinal hemorrhage	Jehovah's Witness	7 units	Died	N	HBOC-201 can adequately serve as initial therapy to maintain tissue oxygen delivery while waiting the maximal effect of recombinant erythropoietin on bone marrow RBC production.
Pachinburava et al. (32)	Case report	Severe Autoimmune Hemolytic Anemia	Due to the presence of allogeneic antibodies and autoantibodies, it extremely difficult to find blood for patients.	2 units	Discharged	Increased methemoglobin	HBOC-201 successfully assisted blood transfusion therapy.
Epperla et al. (33)	Case report	Warm autoimmune hemolytic anemia	Jehovah's Witness	27 units	Discharged	Hypertension, increased methemoglobin, achalasia	Clinicians' early recognition of the patient's needs and familiarity with its characteristics are essential for safe and timely use.
Gomez et al. (34)	Case report	Kidney-pancreas transplant	Jehovah's Witness	12 units	Discharged	Hypertension, increased methemoglobin, pulse oximetry desaturation.	HBOC-201 can be used as a life-saving measure for patients with severe anemia who refuse blood transfusion, and it should be regarded as a bridging alternative until the patient's hematocrit has returned to a safe level.
Davis et al. (35)	Case report series	Severe sickle cell crisis	Jehovah's Witnesses or compatible RBCs were not available	Two patients received more than 20 units; the other received 27 units	All discharged	Myocardial injury, stroke, achalasia, hypertension, increased methemoglobin	HBOC-201 can provide an oxygen bridge in SCC, until Hb levels are restored to meet metabolic demands.
Epstein et al. (36)	Case report	Sickle cell anemia	Hyperhaemolysis syndrome	One unit	Discharged	Bilirubin to 8.2 mg/dL and ALT to 218 U/L (might laboratory effect)	Multi-drug regimens including bortezomib and HBOC-201 have been successfully used in an extreme condition of HHS.
Unnikrishnan et al. (37)	Case report	Sickle cell disease	Anti-N and anti-Doaimmunoglobulin G alloantibody-mediated delayed hemolytic transfusion reaction with hyperhaemolysis	19 units	Discharged	Hypertension, increased methemoglobin and liver enzymes	HBOC-201 can be a lifesaving alternative in this hyperhemolysis. This study also further supported the use of eculizumab in severe DHTR.
Zumberg et al. (38)	Case report series	Severe anemia	Life threatening anemia	At least 10 units of HBOC-201 (*n* = 10)	All discharged	Increased methemoglobin, gastrointestinal symptoms, hypertension	For patients with severe anemia who cannot be infused with RBC, long-term use of HBOC-201 is a feasible and safe oxygen bridge.
Donahue et al. (39)	Case report	Acute lymphoblastic leukemia	Refused allogeneic blood transfusions	15 units	Discharged	Increased methemoglobin	When blood transfusion is not an option, HBOC-201 can be used as a life-saving intervention for patients with severe anemia.
Agrawal et al. (40)	Case report	Relapsed secondary acute myeloid leukemia	Jehovah's Witness	1,230 g (41 units)	Died	Renal toxicity	The homogeneous extraction of oxygen by the brain in the presence of and perhaps from HBOC-201 was demonstrated.

*N: not reported*.

## Properties of HBOC-201

Hemoglobin-based oxygen carrier-201 is derived from bovine RBCs. Free Hbs are then purified by chromatography and cross-linked with glutaraldehyde to increase its stability and molecular size. Unlike human RBCs, HBOC-201 has no cell membrane, no blood type, no cross-matching requirement, no risks of viral infection, a long-term storage capability, timely availability, and other characteristics (Table 3). The molecular structure of HBOC-201 is similar to that of human Hb and the molecular diameter of HBOC-201 (8 nm) is much smaller than the RBC diameter (7,000 nm), which can pass through narrow blood vessels where RBCs cannot pass and penetrate the subendothelial space (41). Since HBOC-201 has a lower oxygen affinity than human Hb, it increases the rate of oxygen uptake. As the Hb dissociation curve shifts to the right, the release of oxygen to the tissue is promoted (42). HBOC-201 use chloride instead of 2,3-diphosphoglycerate (2,3-DPG) as on-loading and off-loading of oxygen (43). HBOC-201 has lower concentrations of organic phosphates, which leads to facilitated oxygen unloading in ischemic tissue (the acid Bohr effect) and increase Hb binding of carbon dioxide in the deoxygenated state (the Haldane effect) (44). The physiological effect of 1 g of bovine Hb in HBOC-201 is equivalent to 3 g of human Hb. Analysis of oxygen binding and release properties of Hb by Gregory et al. showed that HBOC-201 could theoretically deliver more oxygen to tissues than RBCs under physiological conditions (16). The results of Thomas et al. also suggested that an increase in extracellular Hb concentration increased oxygen transport efficiency for both the uptake and release (42). The plasma clearance rate of HBOC-201 follows first-order pharmacokinetics, regardless of whether it is a single dose or multiple doses, which intravascular half-life is 19 h (45). The metabolism of HBOC-201 in men and women seems to be similar and metabolized by the mononuclear phagocyte system (MPS) to bilirubin. Ashenden et al. reported that after healthy male subjects were infused with HBOC-201, the blood oxygen-carrying capacity was improved (46). HBOC-201 has been shown to be effective in increasing oxygen extraction and delivery to peripheral and central organs and improving tissue oxygenation in studies related to isovolumetric hemodilution (47–51). Although HBOC-201 can induce the production of immunoglobulin G (IgG) antibodies in humans, it will not lyse RBCs and its efficacy is not affected by specific IgG antibodies (52). Immunoglobulin E (IgE) anti-HBOC-201 was not detected in the serum samples of patients who received repeated HBOC-201 treatment (53). Therefore, repeated infusions of HBOC-201 appear to be safe for patients and do not increase the risk of type 1 hypersensitivity.

**Table 3:** Overview of the characteristics of HBOC-201 and human red blood cells.

**Characteristics**	**HBOC-201**	**Human red blood cells**
Hemoglobin	Bovine-derived, glutaraldehyde polymerized	Human-derived
Average molecular weight	250 KDa	64 KDa
Molecular diameter	8 nm	7,000 nm
PH	7.6–7.9	7.35–7.45
Ion concentration	Na^+^: 145–160 mmol/L, Cl^−^: 105–120 mmol/L, K^+^: 3.5–5.5 mmol/L,Ca^2+^: 0.5–1.5 mmol/L	Na^+^: 135–150 mmol/L, Cl^−^: 96–106 mmol/L, K^+^: 13.5–5.5 mmol/L, Ca^2+^: 2.25–2.75 mmol/L
Osmolarity	290–310 mOsm/L	280–310 mOsm/L
Hemoglobin concentration	13 g/dL	11–16 g/dL
Oxygen bound per gram of hemoglobin	1.36 ml	1.34 ml
Methemoglobin concentration	<5%	<2%
P50	43 mmHg	27 mmHg
Viscosity (37°C)	1.3 centipoise	4 centipoise
Temperature range of application	4–37°C	37°C
Half life	19–24 h	120 days
Shelf life	3 years	3 weeks

## Trauma and Hemorrhagic Shock

After trauma, HS can lead to insufficient perfusion of vital organs, cell hypoxia, and organ dysfunction. HBOC-201 can provide sufficient oxygen, reduce the risk of disease transmission, and longer shelf-life. Its effect on neutrophil activation and immune function is similar to other resuscitation fluids, suggesting that it may be safe as a resuscitation fluid in patients with HS (54, 55). Compared with various resuscitation fluids, HBOC-201 can restore tissue oxygenation, maintain blood pressure, reduce the need for blood transfusion in the hospital, reverse anaerobic metabolism, and even improve survival rate (56–59). Even for low-volume resuscitation, HBOC-201 still can improve intracranial pressure and brain oxygen (60–62), improve hemodynamics (63), avoid blood transfusions (64), and improve survival rate (65). Although some preclinical studies have elevated aspartate aminotransferase in the early stage of HBOC-201 infusion, they tend to be normal afterward (63). Incidentally, the usage of HBOC-201 during HS resuscitation, combined with physiological parameters and hypotension to guide fluid volume, will reduce the risk of hyperinfusion and increase the potential benefits. The low-volume strategy for HS resuscitation can maximize the beneficial effects of HBOC-201, while minimizing any potentially harmful effects.

Trauma, bleeding, and fluid resuscitation will cause the consumption and dilution of coagulation factors, adversely affecting the coagulation of trauma patients. Arnaud et al. in a preclinical study showed that transfusion of HBOC-201 successfully resuscitated and avoided blood transfusion in animals, but it caused dilutional coagulopathy (lower hematocrit, platelets, and fibrinogen) (66). HBOC-201 combined with other coagulation substances such as freeze-dried plasma (FDP) (67) or recombinant coagulation factor VIIa (rfVIIa) that had little effect on functional coagulation indicators and had no significant survival benefit (68). However, in the combination of a bleeding model with the Folts model, due to the interaction of many factors such as platelet count, hematocrit, and platelet aggregation, HBOC-201 can reduce thrombosis (69).

In a porcine model with controlled bleeding (40%) and soft-tissue injury, infusion of HBOC-201 not only improved tissue perfusion, stabilized hemodynamics, reduced fluid requirements, and mortality (70), but also increased strong ion difference (SID) and electrochemistry to promote alkalosis (71), which might be beneficial for therapy in high lactate conditions. In the more severe porcine model with rectus abdominus crush (64), the benefit from HBOC-201 might become more pronounced. In a porcine model of uncontrollable bleeding caused by liver injury, HBOC-201 stabilized tissue oxygenation, stabilized hemodynamic and metabolic parameters, and improved survival (59, 66, 72–74). Although Gurney et al. reported that HBOC-201 increased systemic and pulmonary artery pressure (59) and Johnson et al. found that aspartate aminotransferase, lactate dehydrogenase, alkaline phosphatase, and mild renal papillary damage in the HBOC-201 group were higher than those of 6% hetastarch (HEX) (74) and these AEs were mild. In the abovementioned severe HS porcine model, selective aortic arch perfusion (SAAP) and HBOC-201 not only quickly restored survival of cardiovascular function after cardiac arrest (75), but also effectively induced return of spontaneous circulation (ROSC) and provided the critical oxygenation and perfusion support necessary for successful resuscitation (76, 77).

Compared with other resuscitation fluids, HBOC-201 can improve the mean arterial pressure (MAP), cerebral perfusion pressure (CPP), brain tissue oxygen tension [PbtO(2)] and reduce the secondary damage, and the need for blood transfusion in the traumatic brain injury (TBI) model (78–80). In preclinical studies of TBI, infusion of HBOC-201 not only rapidly restored the self-regulatory mechanisms of cardiovascular system, but also improved cerebral blood flow (CBF), CPP, PbtO(2), and cerebral vasoreactivity to hypercapnia (CVH) (78, 81–83). However, the decrease in nitric oxide (NO) caused by infusion of HBOC-201 may cause constriction of the cerebral vasculature. Cerebral metabolic demand for oxygen is critical in the case of TBI and oxygen supply is increased to minimize ongoing neuronal damage and, thus, reduce morbidity and mortality, so cerebral vasoconstriction may have a devastating effect on patients. Since HBOC-201 causes slight vasoreactivity and has the ability to carry and release oxygen efficiently, it may further improve CBF, CPP, and PbtO(2). In the porcine multiple injury model of TBI bleeding and aortic tear injury, the fluid resuscitation of HBOC-201 is not better than that of Lactated Ringer's (LR) solution (82). The possible reason is that in the case of high-pressure aortic injury, the boosting effect of HBOC-201 may lead to increased bleeding and it may also be related to the severity of shock at the beginning of resuscitation. Therefore, when considering the resuscitation strategy, it is necessary to consider the different injuries of high-pressure blood vessels and low-pressure solid organs and the different severity of shock. In future studies of traumatic HS, how to balance the benefits of HBOC-201 infusion with the potential side effects and how to personalize emergency treatment assistance to patients need to be thoroughly investigated in further studies. Jonathan et al. first used HBOC-201 in a patient with severe TBI, a Jehovah's witness who refused a blood transfusion (29). The patient was transfused with 6 units of HBOC-201 (180 g) and non-invasive cerebral oximetric devices were used to monitor the brain tissue oxygen saturation of the patient. The results of this study showed that not only brain tissue oxygen saturation, central venous oxygen saturation, and hemodynamic variables increase significantly after HBOC-201 administration, but total Hb increased from 3.2 to more than 7 g/dl. The patient developed severe hypertension 12 h after the last HBOC-201 injection, followed by massive cerebral edema and death. The possible cause was massive reperfusion injury from delayed repayment of cerebral oxygen debt in a severely ischemic brain, but it cannot yet be excluded that HBOC-201 itself directly caused massive edema and brain herniation. There is no doubt that HBOC-201 rapidly corrected cerebral venous and central venous oxygen saturation, but further applications in patients with TBI and controlled research need to be explored in the future.

## Anemia

Hemoglobin-based oxygen carrier-201 has been allowed by the FDA for use under the expanded access in Jehovah's Witnesses patients who require blood transfusion. HBOC-201 has no cell membrane and lacks cell surface antigens, making HBOC-201 an ideal substitute for blood to save lives when blood is unavailable or blood transfusion is refused for patients with severe autoimmune hemolytic anemia (30, 32, 33, 36, 37). Epperla et al. demonstrated that early recognition of patient needs and familiarity with the characteristics of HBOC-201 by clinicians are essential for the safe and timely use of HBOC-201 to save the lives of the patients (33). Davis et al. reported three cases of critically ill patients with sickle cell disease with multiorgan failure who refused RBCs or were not able to use RBCs and whose lives were ultimately saved by the administration of HBOC-201 therapy (35). This study demonstrated that HBOC-201 could provide an oxygen bridge in treating patients with sickle cell crisis (SCC) until Hb levels restored to meet metabolic demands. A Jehovah's Witness patient with acute lymphoblastic leukemia (ALL) developed severe anemia (Hb as low as 3.1 g/dl) during chemotherapy. During the infusion of HBOC-201, Hb of the patient remained between 3.6 and 5.3 g/dl without any ischemia or organ dysfunction (39). Gomez et al. reported for the first time that kidney–pancreas double solid organ transplant recipients whose Hb dropped to a critical level (2 g/dl) and Hb increased to 6.8 g/dl after infusion of HBOC-201 (34). Gannon et al. demonstrated that a combination of HBOC-201 and high-dose recombinant human erythropoietin (rHuEpo) for life-threatening anemia (Hb, 3.5 g/dl) improved survival of the patient and a significant increase in Hb to 7.6 g/dl (31). This study demonstrated that HBOC-201 can provide adequate tissue oxygen supply until marrow RBC recovery and HBOC-201 combined with high-dose rHuEpo also increase hematocrit and survival in patients with severe symptomatic anemia. Therefore, HBOC-201 is a viable and safe oxygen bridge for patients who cannot infuse RBCs or for whom RBCs are not feasible, hence saving the life of the patient in a life-threatening situation. Furthermore, the existing data supported that although HBOC-201 can cause common side effects such as methemoglobin, gastrointestinal symptoms, and hypertension when the cumulative dose reaches 10 units, it is generally a feasible and safe “oxygen bridge” (13). This study showed that early use of HBOC-201 increased the chances of survival in patients with acute bleeding and hemolysis. Zumberg et al. reported on the treatment of 10 patients with life-threatening anemia and poor prognosis with at least 10 units of HBOC-201 and all of them survived and discharged without long-term complications (38). The results of these cases supported that long-term and high-dose administration of HBOC-201 is a feasible and safe treatment modality for early and proactive correction of life-threatening severe anemia until adequate intrinsic Hb is restored, in cases where blood transfusion is not an option. Agrawal et al. reported on a Jehovah's Witness who presented with chemotherapy-induced anemia following a relapse of secondary acute myeloid leukemia, documenting the highest dose and longest duration of experience with HBOC-201 treatment to date (40). Over a period of 18 days, the patient was infused with 1,230 g of HBOC-201. This study revealed by PET scan that the bilaterally similar and homogeneous extraction of oxygen in the presence of soluble HBOC-201 by the brain tissue. However, nephrotoxicity of HBOC-201 during the infusion of the patient cannot be excluded and, therefore, HBOC-201 may not be useful as the sole oxygenating component of anemia treatment during myelosuppressive therapy. These studies support the safety and feasibility of HBOC-201 in patients with anemia, but the consequences of AEs that occur to patients are worthy of attention. Future randomized, multicenter, and large sample size clinical trials are needed to obtain further high-quality evidence to validate the efficacy, safety, and tolerability of HBOC-201.

## Myocardial Infarction

Since the diameter of HBOC-201 is smaller than that of RBCs, it is easily transported through plasma to places where RBCs cannot reach. Therefore, its characteristic of carrying and releasing oxygen makes HBOC-201 more beneficial than whole blood in emergencies. Infusion of HBOC-201 30 min before myocardial ischemia, the infarct size/area at risk (Inf/AAR) was significantly reduced (84). George et al. also reported a similar study that infusion of HBOC-201 after 15 min after ischemia can also significantly reduce Inf/AAR and improve myocardial survival (85). Bloodless reperfusion is a promising strategy that restores oxygen delivery and delays the re-exposure of ischemic myocardium to blood cells, plasma proteins, and other blood-borne inflammatory substances. Intracoronary preoxygenated HBOC-201 can reduce changes in infarct size and stroke volume, improve aerobic metabolism, systolic shortening (SS), and stroke volume in swine during coronary artery occlusion (CAO) in a dose- and temperature-dependent manner (86). Preoxygenated HBOC-201 can match the oxygen delivery of blood in almost equal amounts. The study by Garciá-Ruiz et al. found that the preoxygenated HBOC-201 solution did not lead to increase in infarct size (IS) or deterioration of long-term ventricular function, but it is still feasible and safe (87). The infarct size in the MI model is larger than other studies, mainly due to the use of intravenous anesthetics to avoid the known infarct-limiting effects of inhaled gas anesthetics. With the introduction of percutaneous coronary intervention (PCI), the morbidity, mortality, and disability rates of acute coronary syndromes (ACSs) have been greatly reduced, but the selection of the best drug to limit myocardial injury occurs during ischemia and early reperfusion remains challenging. Since HBOCs have the ability to deliver oxygen, they have been considered for the treatment of ACS. In a randomized, double-blind study, Serruys et al. made the first attempt to introduce HBOC-201 in ACS treatment, examining the safety and tolerability of HBOC up to 230 ml in low-to-moderate risk cardiac patients scheduled for elective PCI (19). The results showed that although intravenous HBOC-201 had side effects such as cell-free plasma Hb and increased blood pressure, no impaired coronary blood flow or left ventricular stroke work index (LVSWI) was observed in patients, nor did it affect the autoregulation of coronary blood flow. In a single-center, phase II, placebo-controlled, crossover, single-blind study, intracoronary infusion of preoxygenated HBOC-201 in five patients undergoing elective PCI improved myocardial “oxygenation” and maintained left ventricular function during brief coronary occlusion (20). This study demonstrates that HBOC-201 can be effective as an oxygen bridge to preserve myocardial mechanical and electrical properties in the face of total coronary occlusion, prolonging the “golden” time period in patients with PCI and improving myocardial oxygenation in patients with MI. More complex patient populations, including ST-elevation MI (STEMI), can benefit from oxygenated HBOC-201 in the future that remains to be further explored. Moreover, higher quality evidence is needed to further collect and validate the efficacy, safety, and tolerability of HBOC-201 in patients with MI.

## Cardiopulmonary Bypass

Cerebral ischemia may occur during extracorporeal circulation, especially at low flow. Neuronal death due to cerebral ischemia can cause a series of irreversible complications. Jeffrey et al. first verified that in a porcine model of normothermic low-flow cardiopulmonary bypass (CPB), compared with donor whole-blood priming, adding HBOC-201 to the pump-priming solution during CPB seems to improve cerebral oxygenation by minimizing overall end-organ ischemia (88). Cerebral oxygenation was maintained in the HBOC-201 group, even during the decrease in blood flow. Although myocardial impairment and elevated troponin levels following HBOC-201 treatment need to be further evaluated before clinical application, this study supported the possible benefits provided by HBOC-201 during CPB. Extracorporeal membrane oxygenation (ECMO) is an accepted treatment for resuscitating critically ill neonates and children with high predicted mortality. Since the blood volume of children is so small relative to the initiation volume of the ECMO circuit, an extracorporeal circuit containing a large volume of blood is required to initiate the ECMO circuit. The donor RBCs in ECMO circuits carry the risk of viral infection, immune response, and blood from multiple donors. Given these concerns, the use of an effective blood substitute during ECMO would be beneficial for the management of ECMO in child patients. In models of ECMO of piglets with healthy or acute respiratory distress syndrome (ARDS), the results of Gregory et al. and Henderson et al. demonstrated that although the hematocrit of the HBOC-201 group was significantly lower than the blood group and the blood pressure and methemoglobin concentration in the HBOC-201 group were slightly increased, HBOC-201-primed ECMO was well-tolerated, maintained hemodynamic stability, and provided good oxygen delivery (89, 90). HBOC-201-primed ECMO appears to be very promising and feasible. HBOC-201 not only has a long half-life, but also eliminates the risk of infection of the patient, immune response, and exposure to multiple donors. However, the initial dosing, the circuit transfusion support, the potential concerns with respect to methemoglobin and vasoconstriction, and the impact on platelet and coagulation factor depletion still warrant further investigation.

## Perioperative Period

Perioperative preoperative sampling, surgery-related blood loss, and hemodilution result in acute postoperative anemia, which can lead to a reduction in oxygen-carrying capacity. In addition, some emergency surgeries do not have sufficient time and physiologic reserves to donate autologous blood in advance; therefore, many patients require allogeneic RBC transfusions during the perioperative period to maintain cellular energy metabolic needs. During the 1970's and 1980's, many concerns began to be expressed about the safety of blood transfusions. However, a large number of clinical studies have shown that patients after HBOC-201 infusion had good tolerance in different dosing regimens and various clinical conditions, especially in the perioperative period (15, 21, 27, 53). In addition, the improvement of hemodynamics after administration of HBOC-201 may be attributed to its highly colloidal intravascular expansion properties. Although HBOC-201 cannot significantly increase and maintain THb, it can meet the oxygen-carrying needs of patients with severe anemia until RBC is available or patients can establish a safe hematocrit (16). Moreover, in some studies, HBOC-201 can eliminate the need for any allogeneic RBC transfusion for some patients during the perioperative period (22–24). However, insufficient treatment, effective treatment delay, and volume overload were the reasons why the incidence of AE and serious AE (SAE) in the HBOC-201 group was significantly higher than that in PRBC (23).

A multicenter, randomized clinical study showed that HBOC-201 reduced the intraoperative requirements for allogeneic blood transfusion during aortic reconstructive surgery, but led to an increased requirement for allogeneic transfusion later during the hospitalization and ultimately did not reduce the need for allogeneic blood (25). This study indicated that HBOC-201 might be most effective in eliminating need of the patient for emergency transfusions without reducing the total RBC transfusion requirement. In addition, this study discovered that HBOC-201 was associated with a transient increase in systemic blood pressure, but this slight systemic blood pressure increase produced by HBOC-201 may be an advantage for patients with vascular disease who often require temporary blood pressure support. In a study of elective abdominal aortic surgery (26), HBOC-201 provided no significant benefit to hydroxyethyl starch (HES) because the advantage of increased oxygen-carrying capacity was offset by increased vascular resistance and reduced cardiac output. In a limited safety analysis, it can be shown that HBOC-201 is acceptable in the stable trauma, hypotension, and younger age population, especially when safe blood transfusions are not available quickly (91). Overall, in cases where patients require emergency surgery or safe blood transfusions are not rapidly available, HBOC-201 provides a temporary oxygen transport bridge until endogenous RBC mass can be restored to adequate oxygen-carrying capacity or allogeneic transfusion is available.

## Organ Transplantation

For dynamic preservation of isolated organs, the earliest clinical series used is hypothermic machine perfusion (HMP) without oxygenation; however, recent studies have shown that solid organ damage can be mitigated by providing oxygen dissolved in the perfusate (92–94). Subsequent clinical applications for MP employed a single roller pump device utilizing RBCs-based perfusate under normothermic conditions, but it also has several potential drawbacks including the fact that RBCs are a relatively scarce human blood product that may induce immune reactions or cause infections, RBC hemolysis, and logistical difficulties associated with using cross-matched blood (95, 96).

Hemoglobin-based oxygen carrier-201 is a polymerized bovine with low immunogenicity and similar oxygen-carrying capacity to human Hb at normothermic temperatures (21). Compared with cold static preservation (CSP) or blood perfused, the subnormothermic MP (SNMP)/HBOC-201 system significantly improved graft function and provided effective oxygenation to the tissue (97, 98). Laing et al. presented the first experience using HBOC-201 in discarded high-risk human livers of normothermic MP (NMP) (99). The vascular blood flow parameters and lactate clearance rate of the liver perfused with HBOC-201 and RBCs were similar and the HBOC group could extract more oxygen. Compared with RBCs + fresh frozen plasma (FFP) perfused livers, livers perfused with HBOC-201-based perfusion solution had significantly higher hepatic ATP content, cumulative bile production, and portal and arterial flows (100). HBOC-201 was first used in the study of discarded human kidneys NMP, which was similar to PRBC with respect to vascular flow, oxygen consumption, and reconstitution of tissue ATP (101). HBOC-201-*ex-vivo* normothermic limb perfusion (EVNLP) is identical to RBC-EVNLP, retaining muscle contractility and mitochondrial structure (102). The above study showed that HBOC-201 can be used as an alternative oxygen carrier to RBC in livers, kidneys, and isolated limbs NMP. However, White et al. found that RBC + Plasma can minimize injury and provide superior preservation of myocardial function during *ex vivo* heart perfusion (EVHP) compared with HBOC-201-based perfusion solution (100). The reason may be that the experimental conditions of this study promoted the spontaneous oxidation and the production of reactive oxygen species (ROS) of HBOC-201 and the methemoglobin gradually increased.

Red blood cell cannot be used during HMP because the stiffness of the erythrocyte lipid membranes increases and hemolysis occurs at low temperatures, thus prompting the need for an alternative oxygen carrier, especially when combined with hypothermia and NMP (17). Mahboub et al. reported better renal function recovery in a rat donation after circulatory death (DCD) kidney model after rewarming with HBOC-201 compared to rewarming without an oxygen carrier (103). Although continuous cold-to-warm (HBOC-201) protocol is similar to an interrupted hypothermic oxygenated perfusion (HOPE) [University of Wisconsin (UW) solution] + NMP (HBOC-201) protocol in terms of increasing ATP synthesis and reducing tissue expression of oxidative tissue damage markers, the use of a single perfusate eliminated unnecessary ischemia time required for perfusate exchange (103). HBOC-201-based perfusate was used in two clinical studies of the dual hypothermic oxygenated machine perfusion (DHOPE)-controlled oxygenated rewarming (COR)-NMP trial. After being assessed, most livers that met all the criteria were transplanted (17, 28). Totto B et al. increased the number of transplantable livers by 20% by using a combination of sequences DHOPE-COR-NMP and HBOC-201 to resuscitate and evaluate high-risk donor livers (28). Livers of suboptimal quality that are not repaired and functionally assessed post-transplant will increase the risk of primary non-function and early allograft dysfunction in patients. Combining HBOC-201 with the MP platform is feasible and provides novel and safe perfusion protocols for expanding the transplanted organ pool.

## Adverse Events and Resolutions

Hemoglobin-based oxygen carrier-201 was originally developed for use as a blood substitute. Based on the characteristics of HBOC-201, it can increase convective oxygen delivery by increasing the oxygen-carrying capacity of blood and promote the release and diffusive transport of oxygen in the microcirculation, thereby promoting tissue oxygenation. Thus, the application of HBOC-201 can also be extended to various ischemic and hypoxic diseases such as traumatic HS, anemia, surgical settings, MI, CPB, and organ transplantation. The efficacy, safety, and tolerability of HBOC-201 in various clinical scenarios have been shown to be promising in published preclinical and clinical studies. The efficacy, safety, and tolerability of HBOC-201 in various clinical scenarios are promising in published preclinical and clinical studies. Unlike natural Hb, the Hb molecules in HBOC-201 have been chemically altered to minimize such toxicity theoretically. However, several AEs occurring during the application of HBOC-201 require widespread attention, with vasoconstriction and increased blood pressure being of great concern and the highest incidence of AEs. The high affinity of HBOC-201 for NO and the lower shear stress lead to a decrease in circulating NO, which may result in systemic vasoconstriction, decreased blood flow, increased pro-inflammatory mediators and vasoconstrictor factors, and platelet inactivation, ultimately leading to increased MI and mortality in patients (104). The vasoconstriction caused by HBOC-201 may also be attributed to increased release of endothelin (105), stimulation of α-adrenergic receptors (106), and delivery of high oxygen concentrations. HBOC-201-induced vasoconstriction and blood pressure can be modulated by reducing the dose, changing the solvent, and adding a vasodilator. In a porcine model of HS, HBOC-201 suspended in the hypertonic saline (HTS) solution significantly decreased systemic vascular resistance (SVR) and pulmonary vascular resistance (PVR), increased mean pulmonary artery pressure (MPAP) and cardiac output (107), and reduced the high inflammatory response (108). Inhaling NO before intravenous infusion of HBOC-201 can prevent systemic vasoconstriction without causing methemoglobin (18). Sodium nitrite (NaNO_2_) also temporarily reduced systemic and pulmonary blood pressure increase from HBOC-201 in a dose-dependent manner (109). Three vasodilators, sodium nitroprusside (SNP), sodium nitrite and nitroglycerin, can all attenuate HBOC-mediated vasoconstriction, with nitroglycerin being the least reactive with HBOC-201 in methemoglobin formation (110). SNP can effectively and safely reduce HBOC-201-related systemic, but not pulmonary vasoactivity (111). Therefore, among the three nitrovasodilators, nitroglycerin seems to be the most promising drug for reducing hypertension caused by HBOCs.

Increased methemoglobin is another common side effect of HBOC-201 application. Unlike RBCs, HBOC-201 lacks the nicotinamide adenine dinucleotide (NADH)-dependent enzyme (methemoglobin reductase). Excessive methemoglobin will lead to a decrease in the ability to release oxygen, which will reduce the amount of oxygen delivered to the tissues. Iron ions mediate the oxidative damage of endothelial cells, leading to increased microvascular permeability. Methemoglobin concentrations below 10% do not significantly alter the mode of oxygen delivery to organs (112). Juraj et al. found that the percentage of methemoglobin was delayed to increase after HBOC-201 infusion, so the methemoglobin load was not caused by HBOC-201 infusion, but was gradually generated by oxidation of plasma Hb. Therefore, close monitoring of the changes in methemoglobin in patients and timely management are beneficial to minimize the damage caused by high concentrations of methemoglobin. The methemoglobinemia resulting from HBOC-201 infusion in the patient in the case report by Athira et al. responded well to vitamin C and showed no major toxicity secondary to HBOC-201 (37). In addition, Colin et al. showed that the increase in methemoglobin was independent of the dose of HBOC-201 and did not affect patient survival and that patients responded to injectable methylene blue infusions (13). The percentage of methemoglobin can be corrected or reduced by adding additional HBOC-201, glutathione, or vitamin C to the perfusion fluid during the perfusion of isolated organs (17). Since vitamin C affects the pH and osmolarity of the perfusate, addition to the perfusate to reduce methemoglobin is not recommended.

In addition to the two common AEs mentioned above, AEs recorded with HBOC-201 include plasma volume expansion, gastrointestinal discomfort, blood urea nitrogen values, liver and pancreatic enzyme dysregulation, yellow skin, and sclera discoloration, etc. The use of diuretics may improve plasma volume expansion in patients and anticholinergics may treat gastrointestinal discomfort. The increase in blood urea nitrogen may be due to the high protein load associated with the infusion of HBOC-201. A transient increase in hepatic transaminase and pancreatic enzyme concentrations is not usually associated with hepatic or pancreatic dysfunction (19). This may be due to an increase in metabolic load after absorption, distribution, metabolism, and excretion of HBOC-201 involving the hepatopancreatic system (19), resulting in upregulation of enzyme activity or it may be due to very slight damage to hepatocytes and pancreatic cells during the catabolism of HBOC-201. The mechanisms of elevated hepatic and pancreatic enzymes and the clinical significance need to be explored more specifically and in detail in future clinical studies. The skin discoloration and serum discoloration caused by HBOC-201 will not cause problems in pulse oximetry monitoring. The above-mentioned AEs are transient, but close observation and documentation of HBOC-201 in the clinical setting are still required to allow clinical decision-makers to respond promptly with active intervention.

## Conclusion

Various clinical scenarios of ischemia and hypoxia need to be corrected in time to reduce the sequelae and mortality of patients. HBOC-201 has many potential advantages including being easily available, not affected by storage, and the ability to transport oxygen more efficiently than Hb in RBCs, which is currently a more desirable oxygen carrier. A large amount of evidence shows that HBOC-201 can be used as a temporary oxygen bridge, not only can improve the oxygenation of tissues and organs in emergency situations until the RBCs *in vivo* recover enough oxygen-carrying capacity, but also can provide oxygen to ischemic and hypoxic tissues or/and organs to mitigate ischemia-reperfusion injury. Based on the safety considerations of HBOC-201, clinicians would pay attention to monitoring and recording AEs related to cardiovascular, methemoglobin, clinically relevant liver enzymes and pancreatic enzymes, kidney, gastrointestinal disorders, skin and sclera color, etc. Due to different clinical scenarios, the application of HBOC-201 dosage and protocols would be further explored in the future and individualized treatment of patients will be carried out to maximize potential benefits and minimize adverse consequences. In addition, multicenter randomized clinical trials with large sample sizes would be implemented to obtain high-quality evidence to further verify the efficacy, safety, and tolerability of HBOC-201.

## Author Contributions

MC, DZ, and XH conceived the idea, coordinated the staff involved in this study, and wrote the first draft. YZ, HHe, RY, and LP revised manuscript critically for important intellectual content. HHu, YR, QQ, XY, TY, and LM were involved in the data collection and checked the data. DZ and XH were responsible for final edits and general revisions. All the authors have read and approved the final version of the manuscript.

## Funding

This study was supported by the National Natural Science Foundation of China (81470667), the Department of Science and Technology of Sichuan Province (20YFS0435), and the Science and Technology Bureau of Chengdu (YF06-00070-SN, YF05-00198-SN).

## Conflict of Interest

The authors declare that the research was conducted in the absence of any commercial or financial relationships that could be construed as a potential conflict of interest.

## Publisher's Note

All claims expressed in this article are solely those of the authors and do not necessarily represent those of their affiliated organizations, or those of the publisher, the editors and the reviewers. Any product that may be evaluated in this article, or claim that may be made by its manufacturer, is not guaranteed or endorsed by the publisher.
